# Airway and Esophageal Stenting in Patients with Advanced Esophageal Cancer and Pulmonary Involvement

**DOI:** 10.1371/journal.pone.0003101

**Published:** 2008-08-29

**Authors:** Fabrice Paganin, Laurent Schouler, Laurent Cuissard, Jean Baptiste Noel, Jean-Philippe Becquart, Mathieu Besnard, Laurent Verdier, Denis Rousseau, Claude Arvin-Berod, Arnaud Bourdin

**Affiliations:** 1 Service de Pneumologie, GHSR, St Pierre, France; 2 Service de Gastro-entérologie, GHSR, St Pierre, France; 3 Service de Radiologie, GHSR, St Pierre, France; 4 Service de Chirurgie Thoracique, GHSR, St Pierre, France; 5 Service d'Anesthésie-Réanimation, GHSR, St Pierre, France; University of Giessen Lung Center, Germany

## Abstract

**Background:**

Most inoperable patients with esophageal-advanced cancer (EGC) have a poor prognosis. Esophageal stenting, as part of a palliative therapy management has dramatically improved the quality of live of EGC patients. Airway stenting is generally proposed in case of esophageal stent complication, with a high failure rate. The study was conducted to assess the efficacy and safety of scheduled and non-scheduled airway stenting in case of indicated esophageal stenting for EGC.

**Methods and Findings:**

The study is an observational study conducted in pulmonary and gastroenterology endoscopy units. Consecutive patients with EGC were referred to endoscopy units. We analyzed the outcome of airway stenting in patients with esophageal stent indication admitted in emergency or with a scheduled intervention. Forty-four patients (58±\−8 years of age) with esophageal stenting indication were investigated. Seven patients (group 1) were admitted in emergency due to esophageal stent complication in the airway (4 fistulas, 3 cases with malignant infiltration and compression). Airway stenting failed for 5 patients. Thirty-seven remaining patients had a scheduled stenting procedure (group 2): stent was inserted for 13 patients with tracheal or bronchial malignant infiltration, 12 patients with fistulas, and 12 patients with airway extrinsic compression (preventive indication). Stenting the airway was well tolerated. Life-threatening complications were related to group 1. Overall mean survival was 26+/−10 weeks and was significantly shorter in group 1 (6+/−7.6 weeks) than in group 2 (28+/−11 weeks), p<0.001). Scheduled double stenting significantly improved symptoms (95% at day 7) with a low complication rate (13%), and achieved a specific cancer treatment (84%) in most cases.

**Conclusion:**

Stenting the airway should always be considered in case of esophageal stent indication. A multidisciplinary approach with initial airway evaluation improved prognosis and decreased airways complications related to esophageal stent. Emergency procedures were rarely efficient in our experience.

## Introduction

Management of advanced esophageal cancer (EGC) is still a medical and technical challenge [1]. Most of the inoperable patients are unable to swallow food, and therefore undergo intravenous alimentation with related nosocomial complications. In case of esophagotracheal or bronchial fistulas, the immediate prognosis is poor resulting in continuous aspiration, mediastinis and pneumonia [2].

**Figure 1:**
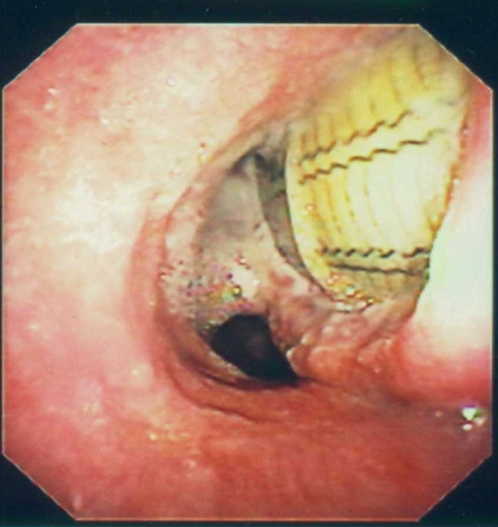
Perforation of the trachea by an esophageal stent. The carina and the main left bronchus were severely injured. The initial fibroscopy performed before esophageal stenting, showed a slight intrinsic compression of the lower part of the trachea and main left bronchus.

**Figure 2:**
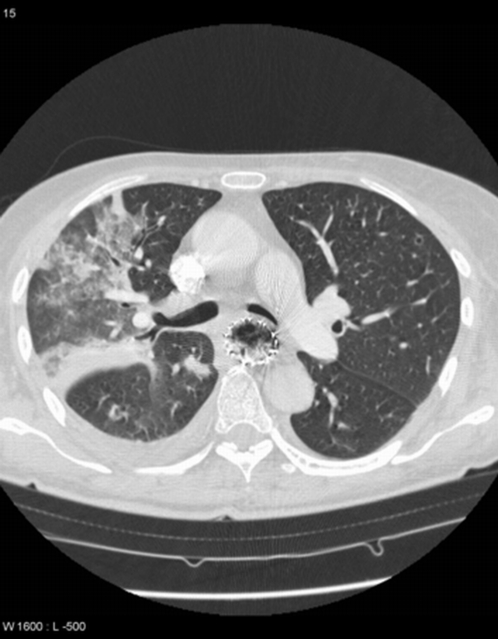
Severe compression of the left main bronchus induced by an esophageal stent. The patient experienced aspiration pneumonia.

Esophageal stenting dramatically improved patients quality of live with a restoration of natural alimentation [3]–[6]. However, in many cases, patients secondarily experienced cough and difficulties in breathing. Acute airway obstruction with asphyxia was described after esophageal stent insertion (figure 1, 2). This complication was related to the esophageal stent protrusion in the airway (figure 1) [7]–[9]. Double stenting has been proposed for the management of esophago-tracheal fistulas [10]–[15].

The aims of this study were to assess the outcome of scheduled and non-scheduled airway stenting, as well as the impact of adequate bronchoscopic evaluation on quality of life and survival in 44 consecutive patients with EGC who were candidate for esophageal stenting.

## Materials and Methods

### Patients

44 consecutive patients (37 males and 7 females, mean age 58±\−8) were analyzed between 2001 and 2007. All patients had a diagnosis of esophageal carcinoma (squamous cells or adenocarcinoma) and were not considered candidates for surgery due to advanced staging. All these 44 patients were indicated for esophageal stenting. All patients were referred to our bronchoscopy department by patient's gastroenterologist. CT scan was initially performed and analyzed. Flexible bronchial fibroscopy under local anesthesia was performed prior to interventional procedure in order to describe airway abnormalities and confirm the indication for an interventional procedure. The latter was the result of a multidisciplinary staff including thoracic surgeon, ENT, digestive endoscopist and an experienced interventional bronchoscopist. Airway findings were classified as follow: 1- esophageal fistulas with trachea and/or main bronchi 2- malignant infiltration of the airway (requiring debulking or not) 3- extrinsic compression of parts of the airway with no malignant infiltration. In general, airways stenting was only proposed if esophageal stent insertion was indicated. In case of fistula and/or malignant infiltration of the airways, a double stenting was systematically proposed. In case of extrinsic compression, an airway stent was proposed in patients with lumen patency lower than 40% or or with bronchial secretion retention below compression. For less severe extrinsic compression, an airway stenting was proposed as a preventive indication to avoid complications induced by the esophageal stent.

### Stent placement

#### Esophageal stent placement

All procedures were performed under general anesthesia. A guide wire was inserted through the fibroscope (Olympus, Tokyo, Japan), across the tumor, and into the distal portion of the esophagus or stomach with fluoroscopic guidance. In severe stricture, a deflated balloon catheter with a 10–12-mm-diameter balloon was then passed over the guide wire to a position across the stricture. The balloon was slowly inflated until the hourglass deformity created by the stricture disappeared from the balloon contour. A metallic covered stent (Ultraflex, Boston scientific, USA) at least 4 cm longer than the stricture was then placed so that its proximal and distal parts rested on the upper and lower margins of the stricture. In case of fistula the covered part of the stent was placed to close the opening. A visual control was performed at the end of the procedure to check the re-opening of the esophagus and/or the fistula closure.

#### Tracheal and/or bronchial stent placement technique

All procedures were performed under general anesthesia with a rigid bronchoscope (EFER, La Ciotat, France). In case of intraluminal malignant proliferation, YAG-laser (Kontron, Eching, Germany) photoablation, or electrocautery (ERBE, Tübingen, Germany) was performed in order to restore airway caliber before airway stenting (Ultraflex, Boston scientific, USA; Dumont stent, Novatech, La Ciotat, France). When needed, a dilation balloon was used to restore airway diameter. The balloon technique was identical as for esophageal stent placement. Prosthetic material was inserted as needed and immediately repositioned under visual control when required as recommended. In case of fistula, the covered part of the stent was placed to cover the opening, and the total length was calculated so that the non-covered bottoms of the stents were in a non-pathologic area of the airway.

#### Timing of both procedures

We tried to insert the airway stent first, to avoid compression of the airway by the esophageal stent. In 13 cases both stents placement were performed during the same procedure, the airway stent first.

### Evaluation and follow-up

All the data were prospectively recorded. Evaluation of airway stenting was performed immediately and within the 1^st^ week after insertion. Follow-up data was collected in 38 patients.

All patients gave a signed inform consent describing the interventional procedure. The study was submitted to and approved by our institution's research committee (Commission de la Recherche Clinique du CHR/GHSR). According to French regulations, no ethic approval was mandatory as patients underwent only standard diagnosis and treatment.

### Statistical analysis

Data are reported in mean+/−standard deviation or percentage. The Mann-Withney U-test was used to assess differences in continuous variable. Clinical factors, therapeutic issues in patient in group 1 vs group 2 were analyzed using the χ2 or Fisher's exact test. Survival difference between sub-group was assessed by Kruskall Wallis test. Variables with a *p*-value less than 5% were considered as statistically significant. Analyses were performed with StatView 2 software, abacus concept, inc, USA.

## Results

Patient's characteristics, general conditions and outcome are listed in table 1 and in table 2. Among the 44 patients, 7 were referred in emergency due to esophageal stent-related airway complications (group 1). Among these 7 patients, 3 of them received a bronchial fiberoptic evaluation prior to esophageal stenting that revealed a fistula (one patient), a slight extrinsic bronchial compression (one patients), and a complex infiltration and stenosis of the carena and the left bronchus (one patient). Time between esophageal stenting and emergency admission for respiratory complications was 8.5+/−9 days (range 2–29). The 37 remaining patients were referred by a multidisciplinary staff for a scheduled stent intervention (group 2). Among the 44 patients, 17 (38.5%) had a fistula, 14 (32%) a malignant infiltration, and 13 (32.5%) an extrinsic compression of the airway. The percentage of lumen obstruction induced by extrinsic compression was assessed by visual evaluation during bronchoscopy, and ranged from 5 to 60% (mean 29+/−12). Fifteen (34%) patients had a tracheal involvement, 17 (38.6%) had a left bronchial involvement. We diagnosed a tracheal, carina, and both main bronchi malignant proliferation in 9 (16%) patients. Three (5%) patients had a tracheal and main left bronchus involvement. A deobstruction procedure was necessary for 16 patients. A single left bronchial metallic stent (16 mm in diameter, 40 mm in length) was inserted in 19 cases, a Y Dumont stent in 4 cases (various adapted size), and a tracheal metallic stent (18 to 20 mm in diameter, 60 to 80 mm in length) in 17 cases. Two patients had a double bronchial stent (Dumont stent for the right bronchus, metallic stent for the left bronchus), and 2 patients a tracheal and left bronchial metallic stent. Airway stenting was impossible for 5 patients.

**Table 1:** Patient's characteristics.

	Number of patients (%) N = 44
Age (years)	58+/−8 years
Gender M/F	37/7
EGC staging	
T4N1M0	30 (68%)
T4N1M1	14 (32%)
EGC therapy	
Radiotherapy	14 (32%)
Chemotherapy	7 (16%)
Radio+chemotherapy	5 (11%)
Best supportive care	18 (41%)
Respiratory symptoms	
Cough	28 (64%)
Dyspnea	19 (43%)
ARF	2 (4.5%)
Aspiration pneumonia	15 (34%)
Miscellaneous†	7 (16%)
Emergency admission	7 (16%)
FOB findings (principal main involvement at the time of airway intervention)	
Fistula	18 (41%)
Malignant	13 (29.5%)
Compression	13 (29.5%)
Time of bronchial stenting*	
Previous	23 (52%)
During same procedure	13 (29.5%)
After	8 (18%)

† Miscellaneous symptoms (fatigue, fever, chest pain).

* Referral time was esophageal stent insertion.

FOB: Fiberoptic bronchoscopy.

**Table 2:** Comparison of demographic data general conditions and cause of death in patients in group 1 and group 2.

	Group 1	Group 2	P value
	N = 7	N = 37	
EGC staging (M0/M1)	3/4	27/10	0.18
Initial performans status			
O–1	4	24	0.69
2–4	3	13	
Respiratory symptoms before esophageal stenting	5/6 (83%)	36/37 (97%)	1
Acute severe symptoms after esophageal stenting	7/7 (100%)	7/37 (19%)*	P<0.0001
Time between stenting and death (weeks)	6+/−7.6	28+/−11	P<0.001
Cause of death			
Immediate airway complications	6/7 (86%)	0	P<0.0001
Late airways complications	0	3/31 (9.5%)	
Pleural effusion	0	4/31 (13%)	
Evolution of EGC	0	20/31 (65%)	
Unknown	0	4/31 (13%)	

MO/M1: presence or absence of EGC metastasis.

* Acute severe symptoms in group 2 are chest pain for 6 patients and a transient shortening of breath for 1 patient.

Complications of the procedure are presented in table 3.

**Table 3:** Interventional procedures.

Airway abnormality	N	Laser or EC	Type of airway Stent§	Severe complication	Non severe complication	Survival (For 38 patients)
**Group 1**	**7**					
Fistula	4	1	T. Metallic: 1	AP: 3	Transient pain: 1	Died 18 w.
		1	Impossible: 3	SS: 1		3 patients died within 1–3 w
Malignant proliferation	1*	1	Impossible	ARF		Died 2 w.
Compression	1		B Metallic: 1	None		Alive (20 w.)
Other/Complex	1†	1	Impossible	AP+ARF+hemoptysis		Died during intervention
**Group 2**	**37**					
Fistula	13	1	T Metallic: 5	Stent obstruction: 2	Transient pain: 7	22+/−11 w
			B Metallic: 6, YDS: 2		Granuloma: 2	2 alive (30 w.)
Malignant proliferation	12	11	T Metallic: 4, B Metallic: 8	Fistula after RT: 1	Transient pain: 8	24+/−9 w.
			DS: 2, YDS: 1		Granuloma: 1	all deceased
Compression	12	0	T Metallic: 7	Migration: 1 Fistula after	Bad breath: 1 Transient pain: 6	27+/−12 w.
			B Metallic: 4, YDS: 1	RT: 1	Granuloma: 1	4 alive (33 w.)

EC: electrocautery. AP: Aspiration pneumonia. SS: septic shock. T: Tracheal. B: Bronchial. DS: Dumont stent. YDS: Y Dumont stent. W.: weeks. RT: Radiotherapy.

§ The number of stents exceeded the number of cases as some patients had multiple stents insertion.

* With ARF (acute respiratory failure) at admission.

† Double fistula of the middle trachea (not related to esophageal stent) and carina. The carina and the 2 main bronchi were destroyed by the esophageal stent. Malignant infiltration was observed. Left bronchus was totally obstructed.

### 1- Esophageal stent complications

#### 1a- in the esophagus

partial opening in 4 patients, non-malignant granuloma at the upper part of the stent in 6 patients (requiring LASER photoablation in 2 cases and, an additional stent within the original esophageal in 4 cases), major throat pain due to a stent inserted for a fistula of the upper part of the esophagus for 1 patient (stent was removed at day 2, and the tracheal stent was efficient alone to close the opening), tumor overgrowth in 2 patients (related to a rapid progression of the disease). Food bolus impaction was easily managed for 2 patients. Stent migration was low and observed in only 2 patients (5.5%).

#### 1b- in the airway

one patient with a left bronchial stent had a non-symptomatic compression due to the esophageal stent. Major complications occurred in the 7 patients admitted in emergency. Five had a protrusion of the esophageal stent in the airway (4 involving the carina) resulting in a major fistula and airway obstruction. One patient had extrinsic compression of the trachea with malignant proliferation requiring urgent LASER deobstruction. One patient had a major compression of the main left bronchus. It was impossible to insert an airway stent for 5 of these patients, who unfortunately rapidly died (table 2).

### 2- Airway stent complications

Airway stents were well tolerated. All patients were asked to perform nebulisation with saline twice a day to avoid airway stent obstruction. Transient pain was observed for 22 (50%) patients. One patient had uncomfortable bad breath. We did not observe any migration for the 26 patients with fistula and esophagotracheal or bronchial malignant proliferation. One preventive bronchial stent migrated after chemotherapy application due to a significant reduction in tumor volume, and was easily removed. Partial bronchial stent obstruction with secretions was observed in 2 cases (easily controlled). A secondary fistula of the carina occurred in 2 patients with double bronchial stents (a Y Dumont stent was inserted after difficult extraction of the metallic stent for 1 patient. No further complication was related for the other one). We observed 4 granulomas at the upper part of the airway stent (1 only required laser therapy, with no relapse).

The impact of stenting is presented in table 4. Respiratory symptoms were improved on day1 and 7 (p = 0.004; RR 3.5: 2–7 and p<0.001; RR 13: 3–55 respectively). Chemotherapy and/or radiotherapy was administered in 26 patients, the over 18 patients had best supportive care. Planned EGC therapy was administrated in 1/5 patient in group 1 and in 25/26 patients in group 2 (p<0.001; RR 20.8: 3–150). Mean survival was 26+/−10 weeks (range: 1–54). Survival was significantly shorter in group 1 patients (6+/−7.6 weeks; range1–22) than in group 2 patients (28+/−11 weeks; range 2–54)) (p<0.001). In group 2, we did not observe survival differences between the 3 sub-groups (fistula, malignant proliferation and, compression). To date, 6 patients are still alive.

**Table 4:** Impact of airway stenting on respiratory symptoms and, EGC therapy.

	Group 1	Group 2	P value RR (95% CI)
	N = 7	N = 37	
**Day 1**	1 (14%)	28 (76%)	p = 0.004 RR 3.5 (2–7)
**Improvement of respiratory symptoms**			
**Day 7**	2 (28%)	35 (95%)	p<0.001 RR 13 (3–55)
**Candidate for EGC therapy**	5 (71%)	26 (70%)	p<0.001
**Achieved to receive therapy**	1 (14%)	25 (96%)	RR 20.8 (3–150)
**Evolution (weeks)**	6+/−7.6	28+/−11	P<0.001
**Candidate for EGC therapy: (RT+/−CT)**			
Fistula	3 (43%)	9 (24%)	
Malignant proliferation	0	7 (19%)	
Compression	1 (14%)	10 (27%)	
Complex	1 (14%)	-	
**Achieved to receive EGC Therapy** *			
Fistula	0	9 (100%)	
Malignant proliferation	0	6 (86%)	
Compression	1 (100%)	10 (100%)	
Complex	0	-	

* % based on patients who were candidate for therapy. 95% CI: 95% confidence interval.

RR: relative risk. EGC: esophageal cancer. RT: Radiotherapy. CT: chemotherapy.

## Discussion

We report here 44 attempts of double stenting involving both the airway and the esophagus in patients with advanced esophageal cancer (EGC). Double stenting was successfully performed in 39 patients an failed in 5 patients who had all been admitted in emergency. Emergency procedures led to poor outcome while scheduled procedures were associated with a better prognosis. We emphasized the potential value of a preventive airway stenting before esophageal procedures in cases of extrinsic compression.

Stenting the esophagus has dramatically improved the quality of life of patients with end-stage esophageal cancer. While esophageal stent usefulness has been published a decade ago [7]–[16], advanced EGC with airway involvement is still a challenging condition for physicians. There are no current guidelines for esophageal stent procedures advising for bronchoscopy prior to stent insertion. Stridor or wheezing may be induced during esophageal dilation with a bougie or a balloon, therefore suggesting major compression of the airways. However, we think that routine evaluation of the airways by fiberoptic bronchoscopy should be mandatory for the evaluation of EGC.

We observed that emergency procedures were associated with a poor prognosis when compared to scheduled procedures. The 7 patients admitted in emergency were referred to the bronchoscopy suite because of respiratory symptoms occurring immediately or shortly after esophageal stent procedure due to wall injury, protrusion in the airway, or dramatically increased preexisting extrinsic compression. In these cases, the pulmonary interventional procedure was found extremely difficult, resulting in failure of airway stenting in 5 cases. One patient died during intervention and, the 4 others deceased within 3 weeks. There are no possibilities to conduct any trial, neither blinded nor controlled, in these emergency palliative situations. Therefore, airway fibroscopy should be discussed as soon as possible for all EGC patients at the time of CT scan examination or esophageal management.

Fortunately, in most patients, a multidisciplinary approach allowed to schedule bronchial stenting before esophageal stenting. Three main indications were considered: fistula, malignant invasion and extrinsic compression. The latter can be considered as a preventive procedure not reported yet.

In case of confirmed esophagotracheal fistula, an airway placement stent to counteract the compression induced by the esophageal device has already been proposed by several groups [10]–[12], [17], [18]. In these cases we systematically inserted tracheal, bronchial or Y Dumont stents. To our opinion, double stenting present the following advantages in patients with oesphageal fistula: i) prevent the protrusion of the esophageal stent in the airway ii) increase the fistula closure by the airway stent iii) decrease stent migrations as both stents interact with each other.

Malignant invasion of the airway was the second indication considered for double stenting. Initial LASER or electrocautery desobstruction+/−additional mechanical debulking was often needed to restore a satisfactory opening of the airway lumen. In these situations, the mechanical properties of the airway wall were severely compromised. Once again, the insertion of the esophageal stent might increase the airway obstruction. The airway stent was then used to restore a tracheal or bronchial framework. In these cases, double stenting can be considered useful to maintain an acceptable airway and avoid a major risk of fistulisation in cases where chemotherapy or radiotherapy is planned.

**Figure 3:**
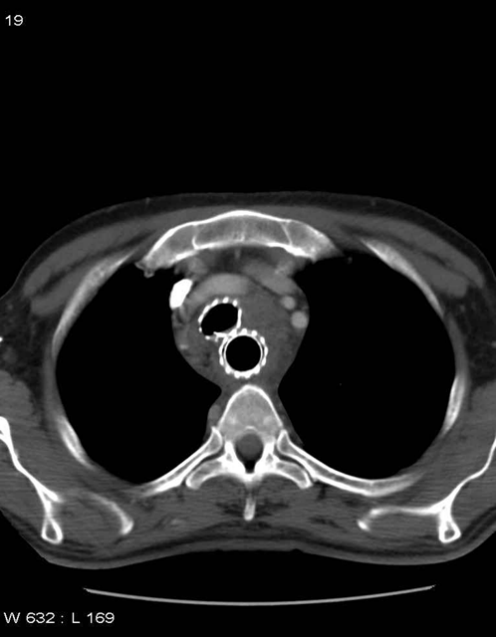
Double stenting in a patient with a voluminous esophageal tumor. Initial staging fibroscopy showed extrinsic compression with no fistula and/or malignant proliferation in the trachea. Esophageal stenting was required because of complete dysphagia. Double stenting was performed before palliative radiotherapy. No complications occurred.

Preventive stent insertion was the third situation. To our knowledge, this is the first report describing this indication. Extrinsic compressions of the airway were the only findings in 12 patients. Due to the asymptomatic nature of these airway compressions we proposed a preventive airway stenting in the narrowed area of the airway before esophageal stenting. It is difficult to predict outcome of an esophageal stent insertion alone. Esophageal stent complications were extensively described in the literature [19]–[28] and, depend on the type of stent used. Older stents (Z stents, wallstents) are more related with complications than modern stents (Ultraflex, polyflex) [27], [28]. Early complications as perforation are variable and ranges from 2 to 20% [22]. Later complications as fistula are also variable (0–10%). Wang et al reported major complications (perforation fistula) in 16% of patients [27] and a review reported up to 30% of these complications [29]. Previous radiotherapy was found to increase the risk of esophageal stent complications [28]–[30]. To our opinion, these complication rates may be underestimated, as airway examination was not reported. In recent reviews, some authors drew attention to the risk of airway compromise when the neoplasic mass narrows the trachea (figure 3) [30], [31]. They advised to consider airway evaluation and tracheo-bronchial stenting in these high risk situations (malignant infiltration in the proximal third part of the esophagus) 22, 30, 31. Perforation and fistula are major and life-threatening complications, and should be avoided in these palliative situations where quality of life is the major end point. We think that preventive double stenting may be considered. Global improvement of respiratory symptoms and, good tolerance of airway stents were major points to promote this procedure. Moreover, scheduled stenting procedure allowed to administer planned EGC therapy for 96% patients, compared to the 14% in the emergency group. This approach may be beneficial to EGC patients. By closing fistulas and decreasing airway complications related to esophageal stenting, chronic aspiration, chronic sepsis, and recurrent atelectasis were uncommon in patients with scheduled airway stenting. Chemotherapy and/or radiotherapy were administered with no delay. Covered metallic stents (ultraflex®) were mostly used because of their high expansion properties to counteract the larger and wider esophageal metallic stent. In case of carina involvement, only Y silicone stents (novatech®) were used. However, all recent marketed stents can probably be considered for this indication depending on physician's experience [32].

The timing of double stenting is the last issue [31]. We tried to insert the airway stent first. As the esophageal stent was always longer and wider, the expansion strength was therefore greater. To stent the trachea several days after the esophageal stenting may be hazardous, and (may) require balloon dilation with a risk of tracheal rupture [33]. In our experience, first stenting of the airway was easy and safe. This should be assessed more systematically.

Our study has several limitations. First, we didn't conduct a controlled study for preventive stents. However, we did not observe any clear difference for prognosis suggesting that preventive airway stenting did not interfere with the general outcome. Blinded, randomised and controlled studies are difficult to conduct in this patient population due to ethical and practical considerations. Second, patient quality of life (QOL) was not assessed by a validated scoring system. However, improvement of respiratory symptoms was a major result of the procedure. As these patients are considered incurable patients on palliative therapy, survival was not considered an appropriate and relevant endpoint. Time between stent insertion and recurrence of symptoms is a better key point. However, end-stage EGC patients have multiple entangled symptoms which makes it difficult to precisely assess symptom recurrence. Moreover, specific GI signs often overshadow respiratory symptoms. Last, we did not clearly evaluate the impact of the esophageal stent on improvement of dysphagia and general conditions (weight gain, recovery of oral intake). However, our results should not be different as indicated in the literature.

In conclusion, our opinion is to consider double stenting in three situations. 1- As a curative approach in case of a fistula and/or malignant airway wall involvement. 2- As a preventive approach in case of an extrinsic tracheal or bronchial compression, even before any symptomatology 3- As a preventive approach in case of a large proximal esophageal tumor when radiotherapy is scheduled. Airway stents must be inserted before or during the esophageal stenting to avoid secondary technical difficulties.

Due to the poor prognosis of EGC, esophageal stenting as a palliative procedure may increase the general QOL. However double stenting (airway and esophageus) may prevent secondary emergency situations with life-threatening complications and recurrent high hospitalization costs.
